# Exploring spatial patterns and hotspots of diarrhea in Chiang Mai, Thailand

**DOI:** 10.1186/1476-072X-8-36

**Published:** 2009-06-24

**Authors:** Nakarin Chaikaew, Nitin K Tripathi, Marc Souris

**Affiliations:** 1 Remote Sensing and GIS field of study, Asian Institute of Technology, Pathumthani, Thailand; 2 Center for Vector and Vector Borne Diseases, Faculty of Science, Mahidol University, Nakhonpathom, Thailand; 3 Institut de Recherche pour le Développement (IRD), Marseille, France

## Abstract

**Background:**

Diarrhea is a major public health problem in Thailand. The Ministry of Public Health, Thailand, has been trying to monitor and control this disease for many years. The methodology and the results from this study could be useful for public health officers to develop a system to monitor and prevent diarrhea outbreaks.

**Methods:**

The objective of this study was to analyse the epidemic outbreak patterns of diarrhea in Chiang Mai province, Northern Thailand, in terms of their geographical distributions and hotspot identification. The data of patients with diarrhea at village level and the 2001–2006 population censuses were collected to achieve the objective. Spatial analysis, using geographic information systems (GIS) and other methods, was used to uncover the hidden phenomena from the data. In the data analysis section, spatial statistics such as quadrant analysis (QA), nearest neighbour analysis (NNA), and spatial autocorrelation analysis (SAA), were used to identify the spatial patterns of diarrhea in Chiang Mai province. In addition, local indicators of spatial association (LISA) and kernel density (KD) estimation were used to detect diarrhea hotspots using data at village level.

**Results:**

The hotspot maps produced by the LISA and KD techniques showed spatial trend patterns of diarrhea diffusion. Villages in the middle and northern regions revealed higher incidences. Also, the spatial patterns of diarrhea during the years 2001 and 2006 were found to represent spatially clustered patterns, both at global and local scales.

**Conclusion:**

Spatial analysis methods in GIS revealed the spatial patterns and hotspots of diarrhea in Chiang Mai province from the year 2001 to 2006. To implement specific and geographically appropriate public health risk-reduction programs, the use of such spatial analysis tools may become an integral component in the epidemiologic description, analysis, and risk assessment of diarrhea.

## Background

Diarrhea is a major public health problem in Thailand. The Ministry of Public Health, Thailand, has been trying to monitor and control this disease for many years. The objective of this study was to analyse the epidemic outbreak patterns of diarrhea in Chiang Mai province, Northern Thailand, in terms of their geographical distributions and hotspot identification. The methodology and the results could be useful for public health officers to develop a system to monitor and prevent diarrhea outbreaks.

Diarrhea is the passage of three or more loose or liquid stools per day, or more frequently than is normal for the individual. It is most commonly caused by gastrointestinal infections (bacterial, viral and parasitic organisms). The infection is spread through contaminated food or drinking-water, or from person to person as a result of poor hygiene [1]. Diarrhea disease is an important cause of morbidity and mortality in many regions of the world, with more than 4 billion cases and 2.5 million deaths estimated to occur annually. [2]. It is widespread all over the world, and especially in developing regions such as Africa, South East Asia and the Eastern Mediterranean, where there is rapid population growth, increased urbanization, and limited safe water, infrastructure, and health systems [1].

In Thailand, diarrhea has been a major public health problem for many years [3-8]. The Bureau of Epidemiology, Ministry of Public Health, estimated nearly 1 million cases every year (in the period 2001–2005: 1,020,377, 1,055,393, 966,760, 1,161,877 and 1,142,581 respectively, with corresponding deaths: 176, 160, 124, 93 and 77). In 2006, the diarrhea incidence was estimated to be 1,245,022 cases and 9 deaths, with the highest incidences occurring in Chiang Mai, Chiang Rai, Khon Kaen and Roi Et provinces, all in the northern and north-eastern region of Thailand.

**Figure 1:**
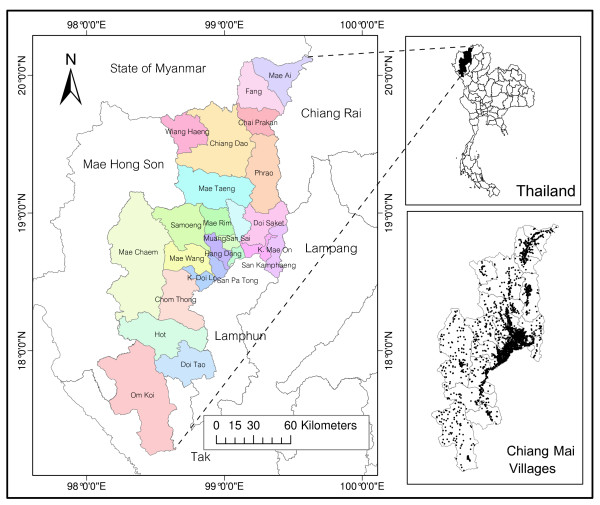
**Chiang Mai, Thailand**.

Chiang Mai is the largest province in northern Thailand, with a geographical location at 18°47'N and 98°59'E (Figure 1), covering an area of 22,061.17 sq. km. It is mostly covered with forested mountain, with an approximate elevation of 310 meters above mean sea level [9]. Chiang Mai province is divided administratively into 24 districts (amphoe), 204 sub-districts (tambon), and 2,070 villages (mooban).

The epidemic pattern of diarrhea in Chiang Mai has fluctuated every year, from 2001 to 2006. The highest number of cases was recorded in 2004, particularly in Doi Tao, Samoeng, and Hot districts, with incidence rates of 6,345, 4,905 and 4,493 per 100,000 people, respectively. The incidence of diarrhea marks high variability at district, sub-district and village levels [10]. Socio-demographic factors (age, education, income etc.), environmental and sanitation factors (poor access to a good water source and poor sanitation) and climate factors (rainfall, temperature and humidity) are thought to be related to incidence and spatial distribution of diarrhea [11,12]. Diarrhea incidence has been increasing in some villages in recent years [10]. A better understanding of the spatial distribution patterns of diarrhea will help to identify areas and populations at high risk and to assist public health officers to plan for control and prevention of diarrhea outbreaks (i.e. the occurrence of a large number of diarrhea cases in a restricted geographical area over a short period of time [13]).

Spatial analyses, such as quadrant analysis (QA), nearest neighbour analysis (NNA) and spatial autocorrelation analysis (SAA), are commonly used to characterise spatial patterns of diseases, and to test whether there is a significant occurrence of clustering of disease in a particular area [14-16]. The quadrant and nearest neighbour methods indicate whether the disease pattern is dispersed, random, or clustered based on counting of incident locations within small squares (QA) and measuring distances to the nearest incident location (NNA) [17-20]. Moran's I and Geary's C are two popular indices of SAA, which are used for detecting spatial pattern of diseases by considering both the incident locations and their attributes (i.e. the disease cases) [21,22]. The local indicators of spatial association (LISA) is a local level test of SAA for identifying whether the incidences of diseases are geographically clustered among the different areas, referred to as "disease hotspots" [23,24]. In this study we researched with the aim to identify spatial patterns of diarrhea based on a hypothesis, which also revealed previously unsuspected patterns leading to the formulation of additional theories. The spatial analyses (QA, NNA and global SAA) were used to investigate spatial patterns of diarrhea incidences. In addition, the LISA was used to indicate the level of spatial autocorrelation that enabled the location of hotspot areas of diarrhea in Chiang Mai during 2001 to 2006.

## Methods

**Figure 2:**
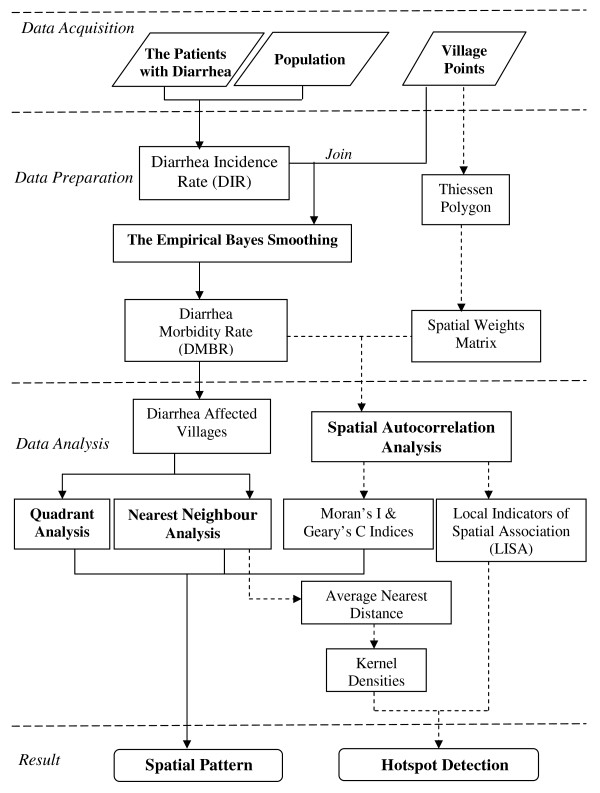
**Flowchart of methodology**.

Methodology is summarised in the flowchart shown in Figure 2:

### Data acquisition

The study of spatial patterns of diarrhea covers the 2,070 villages of Chiang Mai for the period 2001–2006. Data about patients with diarrhea and the population at village level were obtained from the Chiang Mai Provincial Public Health Office (CMPHO), Thailand. These records included the diarrhea cases referred from other hospitals and the population figures from the Ministry of the Interior, Thailand. The spatial data in this study included the village location points in the year 2006, which were collected from the Geo-Informatics and Space Technology Centre (Northern Region) (GISTC), Thailand. All these data were incorporated into a geographic information system (GIS).

Due to different data sources (the number of infected villages from CMPHO was lower than from GISTC), epidemiological data are unknown for some villages (538, 173, 129, 147, 87 and 67 for the years 2001–2006, respectively).

### Data preparation

For each village (i = 1,...,2,070) the diarrhea incidence rate (DIR_*i*_) was defined as the ratio of the number of observed diarrhea cases (*o*_*i*_) to the population (*n*_*i*_):

(1)

Calculating the DIR in this way could lead to spurious spatial features. Villages with small populations could appear highly variable and may contain a disproportionate number of high (or low) parameter estimates. To overcome this problem, an empirical Bayes method based on the idea of pooling information across villages has been developed [24,25]. In this study, these Bayesian principles were used to guide the adjustment of the raw DIR estimate by taking into account information in the rest of the sample. The principle is referred to as shrinkage, in the sense that the raw DIR was moved towards an overall mean, as an inverse function of the variance [26]. The method considers the relative risks as independent and identically distributed, following Poisson distribution with these parameters [27,28]:



Under this assumption, we may obtain, as the estimator of the relative risk for i-th village (), the following expression:

(2)

where *C*_*i *_is the ratio of prior variance to data variance,  is the prior mean (weighted sample mean), and *DIR*_*i *_is the value of the diarrhea incidence rate for each village. The DIR were adjusted by using empirical Bayes smoothing function in the GeoDa software [29], and converted to the diarrhea morbidity rate (DMBR) by multiplying by 1,000.

### Data analysis

#### Diarrhea affected villages

Boxplot was used to represent the spread of the DMBR in Chiang Mai province. It displays the differences between the annual DMBR in the period from 2001–2006 without making any assumptions about the underlying statistical distribution. The spacing between the different parts of the box helps indicate the degree of dispersion (spread) and skew in the data, and identifies outliers or abnormal data [30]. In this study, the diarrhea affected villages (DAV) were classified into 3 categories (low, moderate and high DMBR) by using Jenk's natural breaks classification [31].

#### Spatial pattern analysis

Three different analyses: quadrant analysis (QA), nearest neighbour analysis (NNA) and spatial autocorrelation analysis (SAA), were applied to detect spatial patterns of diarrhea in Chiang Mai province. The village locations and the annualized DMBR at each of these villages were used in the analyses. Both QA and NNA treated only the locations of infected villages but did not distinguish villages by their morbidity rates. Therefore, spatial autocorrelation analysis (SAA) was used to measure and test how villages were clustered/dispersed in space with respect to their DMBR.

The QA requires laying a grid of equally sized quadrants over the region of the study. In our study, the grid we used consisted of 400 quadrants, each with 4.47 km length per side [14]. The variance-to-mean ratio (VTMR) was used in each quadrant to evaluate the level of dispersion of the DAV [18,32-34]. The K-S statistic, which was based upon the variance and mean statistics of a Poisson distribution, was used to test the difference between the observed pattern and the random pattern [34] by setting the significance level as 0.01.

The nearest neighbour index (NNI) used the distance between the DAV in order to evaluate the organization of the spatial distribution of the clusters [35,36]. A Z statistic (with significance level as 0.01) was used to test if the observed pattern was significantly different from a random pattern [37].

The two classic indices of spatial autocorrelation (Moran's I and Geary's C indices [38-41]) were used to evaluate autocorrelation in disease spatial distribution, setting the significance level as 0.01. The indices were evaluated by simulation (9,999 permutation tests).

All these global indices measure the spatial patterns of diarrhea in Chiang Mai province. These indices were performed in GeoDa and ArcView GIS software. The interpretation of the indices values is presented in Table 1.

**Table 1:** Interpretation values for global indices

	**Indices**			
	
**Pattern**	**VTMR**	**NNI**	**Moran's I**	**Geary's C**
Disperse	0 < VTMR < 1	1 < NNI < 2.14	-1 < I < 0	1 < C < 2
Random	VTMR~1	NNI~1	I~0	C~1
Cluster	VTMR > 1	0 < NNI < 1	0 < I < 1	0 < C < 1

#### Hotspot detection

Hotspot is defined as "a condition indicating some form of clustering in a spatial distribution" [42]. This section describes the methods for detecting hotspots of diarrhea by considering both the location of the points (i.e. villages) and their attributes (i.e. the DMBR).

Previous spatial analyses evaluated only global patterns. Local indicators of spatial association (LISA) was used to measure and test spatial distribution (clustered/random/dispersed) at the local level and could be used to determine locations of clusters or hotspots [43]. In this study, local Moran's I value was used to examine the local level of spatial autocorrelation in order to identify villages where values of the DMBR were both extreme and geographically homogeneous [43,44]. It led to identification of so-called diarrhea hotspots, where the value of the index was extremely pronounced across localities, as well as those of spatial outliers. Firstly, the standardized values of DMBR were calculated using the spatial weights matrix that defines a local neighbourhood around each geographic unit [45] and 9,999 permutation tests by setting the significance level as 0.01. Secondly, a Moran scatterplot was produced with a spatial lag of DMBR on the vertical axis and a standardized DMBR on the horizontal axis. Once a significance level was set, values could also be plotted on a map to display the specific locations of hotspots: locations with high values with similar neighbours (high-high) and potential outliers [46].

In order to compare hotspot locations with disease spatial distribution, kernel density (KD) interpolation was used to create a continuous surface representing the density of DMBR across the study area [47-49].

## Results

### Diarrhea affected villages

**Figure 3:**
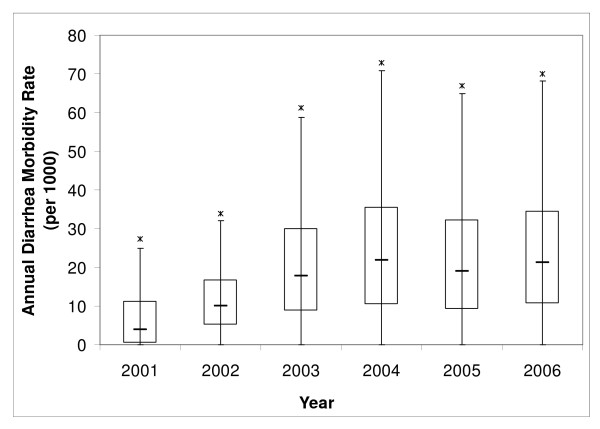
**Parallel boxplots of annual DMBR of 2,070 villages for Chiang Mai province**.

The annual diarrhea morbidity rate (DMBR) in each year (2001–2006) can be presented with parallel boxplots (Figure 3). The boxplots of every year show a positive skewed distribution of annual DMBR; the rate distribution in 2001 and 2002 appear to have smaller variability than the other years. The largest distribution and highest median rate were found in 2004, indicating that the people in this year were more infected with diarrhea than in the other years.

**Figure 4:**
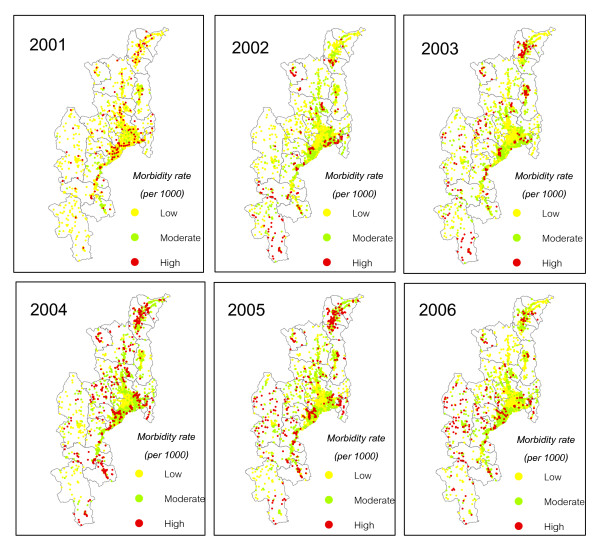
**Diarrhea affected villages during 2001–2006**.

The diarrhea affected villages (DAV) were classified into low, moderate and high-DMBR categories based on the Jenk's optimization method. DMBR values below 20.4 were assigned to the low rate category. Values from 20.04–41.02 were considered moderate rate. DMBR values above 41.02 were classified as high rate. Figure 4 shows villages which were infected with diarrhea from 2001 to 2006. The high risk villages mostly occurred in the north and middle part of Chiang Mai. These were concentrated in Fang district for the years 2003–2005 and spread to Mae Chaem district in the recent years.

### Spatial pattern analysis

Table 2 gives the global indices calculated by using quadrant analysis (QA) and nearest neighbour analysis (NNA). The results show that the spatial distribution of DAV from the years 2001–2006 were clustered (47.29–94.69) according to the variance-to-mean ratio (VTMR), and clustered (0.32–0.52) for the nearest neighbour index (NNI). Table 2 shows the VTMR for the distribution of villages with infected cases in 2004 to be higher, indicating more clustering than that observed for the other years. On the other hand, the spatial pattern of DAV in 2001 was highest clustered for the NNI.

**Table 2:** Global indices for the diarrhea affected villages

	**Indices**		
		
**Year**	**VTMR**	**NNI**	**Pattern**
2001	47.29^a^	0.52^a^	Clustered
2002	67.59^a^	0.34^a^	Clustered
2003	57.64^a^	0.32^a^	Clustered
2004	94.69^a^	0.37^a^	Clustered
2005	65.49^a^	0.36^a^	Clustered
2006	66.73^a^	0.43^a^	Clustered

note a: the corresponding p-value is < 0.01

The global SAA for annualized morbidity rate of villages in Chiang Mai from 2001 to 2006 showed that the Moran's I (0.02–0.40) and Geary's C (0.72–0.95) values were significant (0.01 significance level) for each year (Table 3), implying that distribution of the affected villages with diarrhea was somewhat spatially autocorrelated (low clustered) though the overall tendencies were not so strong.

**Table 3:** Global indices of spatial autocorrelation

	**Indices**		
		
**Year**	**Moran's I**	**Geary's C**	**Pattern**
2001	0.02^a^	0.95^a^	Clustered
2002	0.38^a^	0.79^a^	Clustered
2003	0.32^a^	0.75^a^	Clustered
2004	0.40^a^	0.77^a^	Clustered
2005	0.39^a^	0.76^a^	Clustered
2006	0.40^a^	0.72^a^	Clustered

note a: the corresponding p-value is < 0.01

The VTMR and NNI values were used to demonstrate that villages with cases were not randomly distributed in space among all villages in the province. On the other hand, global Moran's I and Geary's C indices measured the autocorrelation in the incidence ratio among all villages, including villages with low DMBR or no cases, which were therefore less sensitive to clustering.

### Hotspot detection

**Figure 5:**
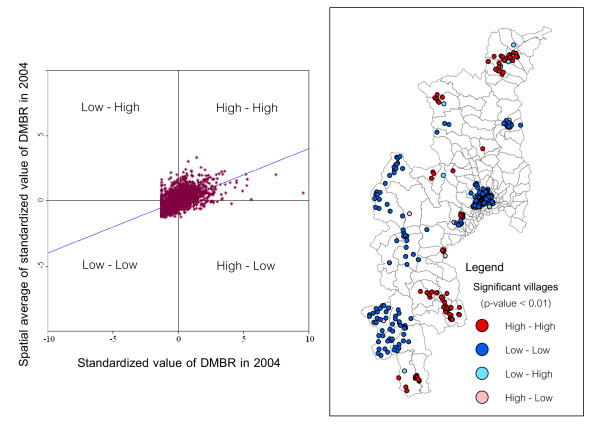
**Moran scatter plot matrix and LISA cluster map of DMBR for p < 0.01**.

The map in Figure 5 shows the locations with significant local Moran statistics and classifies those locations by type of association (LISA cluster map). The outputs from LISA represent the spatial autocorrelation of diarrhea incidence at the village level. The study only focused on the univariate spatial distribution and the location of any significant clusters or spatial outliers in the DMBR data. On the right hand panel of Figure 5, the sample LISA cluster map of DMBR in 2004 is shown, depicting the locations of significant local Moran's I statistics, classified by type of spatial association. The dark red and dark blue locations were indications of spatial clusters (respectively, high surrounded by high, and low surrounded by low). In contrast, the light red and light blues were indications of spatial outliers (respectively, high surrounded by low, and low surrounded by high). There were some outstanding spatial clusters of DMBR covering specific areas in 2004. The clustered villages with high DMBR (hotspots) were found to cover the conurbations in the north and south of Chiang Mai (Fang and Doi Tao districts). In the left hand panel of Figure 5, the corresponding Moran scatterplot is shown. The standardized values of DMBR in each village are first displayed in spatial scatterplot to contrast observed values with their spatial average (spatially averaged adjacent values) and to detect outliers by obtaining the significance level as 0.01.

**Figure 6:**
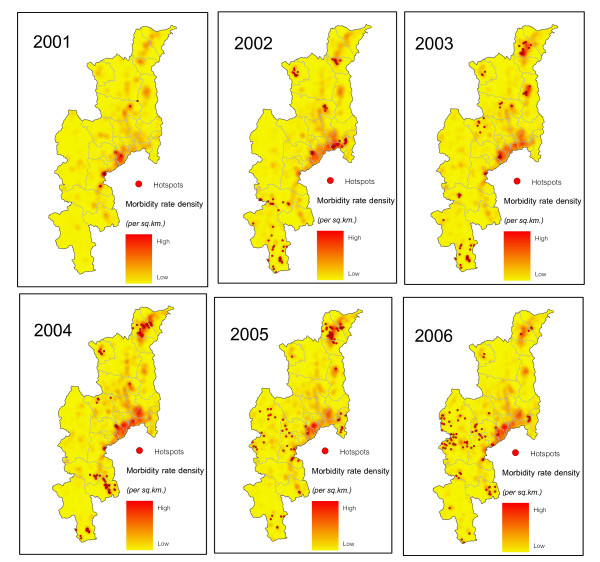
**Hotspots of diarrhea during 2001 – 2006**.

The hotspots of diarrhea during 2001–2006 were found and illustrated by overlaying the kernel density maps, as shown in Figure 6. The maps of local spatial correlation indices were used to display the hotspots with red coloured points (respectively, high surrounded by high). These maps show clear spatial patterns of diarrhea that were concentrated in the middle (San Kamphaeng district), north (Fang, Chai Prakan and Wiang Haeng districts) and south (Doi Tao district) of the study area during 2001–2005 while in 2006 they were mostly spread in the west (Mae Chaem district) of Chiang Mai. The densest clustering of hotspots occurred within the urban areas of San Kamphaeng district in 2002, Doi Tao district in 2004 and Fang district for the years 2003–2005. For the years 2005–2006, the hotspots were distributed around the highland of Mae Chaem district, the resident area of the minorities (Hmong, Karen and Lisu) in Chiang Mai [50].

## Discussion

Estimates of diarrhea incidence rates (DIR) accounted for the variability in population distribution. The Bayesian smoothing technique addresses the issue of heterogeneity in the population at risk, and it is therefore recommended for use in explorative mapping of disease/incidence rates.

The study showed that spatial distribution patterns of diarrhea were significantly clustered, and identified the diarrhea hotspots in Chiang Mai. KD estimation illustrated variation in the grouping of diarrhea locations across the study area, and strongly confirmed the visible pattern on the point location map. From 2001 to 2006, we can see that hotspots migrated from urban villages to highland villages, which have had limited safe water, infrastructure, and health systems [50] in recent years. The spatial distribution of diarrhea is always correlated with socio-demographic factors, and with environmental, sanitation, and climate factors [11,12]. It would be helpful to investigate the underlying socio-demographic or environmental causes of high incidence areas and hotspots identified in this study.

However, there were some limitations in the study:

1) As mentioned before in the data acquisition paragraph, due to different data sources, epidemiological data were unknown for some villages, which were either new or re-structured and therefore did not figure in older data from other sources. The incidence rate for such cases was set to 0. This might further bias research while comparing different years of diarrhea incidence. For example, 2001 shows a lower spatial cluster pattern of affected villages than the other years. This is quite natural as some villages did not exist at that time.

2) The number of patients with diarrhea was classified by year and by village, but not by socio-demographic characteristics (age, gender and education). Although these characteristics can be an important determinant for diarrhea diseases (for example, diarrhea occurs mainly in children under five years of age [11]), they were not available in the epidemiological reports and were not included as a determinant in this study.

## Conclusion

Using spatial analysis methods in GIS, we explored the spatial patterns of diarrhea in Chiang Mai from 2001 to 2006. We show that the spatial distribution is clustered, which areas of diarrhea epidemic were densely clustered, and highlight the spatial trends of the hotspots in the province.

This study exhibits that these methods and tools can be useful for diarrhea surveillance for public health officials. It demonstrates that using existing health data, spatial analysis and GIS can provide an opportunity to specify the health burden from diarrhea within infected areas, and also lay a foundation to pursue further investigation in correlated factors responsible for increased disease risk. To implement specific and geographically appropriate risk-reduction programs for public health officers, the use of such spatial analysis and tools should become an integral component in the epidemiologic description and risk assessment of diarrhea.

## Competing interests

The authors declare that they have no competing interests.

## Authors' contributions

NC, NKT, and MS collaborated intensely on research design and data analysis. NC carried out most of the data preparation, and the spatial and statistical analysis. NC and NKT worked on manuscript preparation. NC, NKT and MS interpreted the data, intensively revised the manuscript, and provided important intellectual discussions. All authors read and approved the final manuscript.
